# Difficulties Limiting Access to Sports and Recreational Facilities in the City in the Perceptions of Service Users. Sports and Recreational Infrastructure Management Policy—Poznan Case Study

**DOI:** 10.3390/ijerph17051768

**Published:** 2020-03-09

**Authors:** Ewa Kruszyńska, Joanna Poczta

**Affiliations:** 1 Faculty of Health and Physical Education, The University of Szczecin, 70-453 Szczecin, Poland; ewa.kruszynska@usz.edu.pl; 2 Faculty of Health Sciences, Poznan University of Physical Education, 61-871 Poznan, Poland

**Keywords:** access to sports and recreation facilities, sports and recreation offer, sport participation, sports and recreation infrastructure, sports and recreational activities, physical activity, healthy lifestyle

## Abstract

The range of sports and recreation facilities’ offer should be very wide in order for all social groups of the examined environment to have access to sports and recreation facilities. Therefore, Poznan City Hall should take into account all difficulties that limit the access to these facilities when preparing management policy of sports and recreation infrastructure and its functioning. That is why the main goal of this study is to recognize difficulties limiting the access to sports and recreation facilities in this city. The research carried out at indoor tennis courts (16 facilities), indoor swimming polls (12 facilities) and fitness clubs (11 facilities) in Poznan covered 1159 service recipients (using the services of a given sports and recreation facility). The author-constructed questionnaire addressed to the service recipients (residents of the city of Poznan who use the services offered at the examined sports and recreation facilities). Furthermore, the service recipients were divided into a group of people doing sports competitively and a group of recipients who have never practiced sports as professional athletes. The validated questionnaire sent to the examined service recipients included suggestions of difficulties that may limit the access to sports and recreation facilities in the city of Poznan. Further analysis of the importance of difficulties that limit the access to sports and recreation facilities may allow indicating the reason why recreational activity of residents is limited. A lower level of satisfaction and thus a lower rating of the respondents was obtained by a set of features constituting, according to the respondents, difficulties limiting the access to the use of sports and recreation facilities (high prices and too great of a distance of the sports and recreation facility from the place of residence).

## 1. Introduction

### 1.1. Contemporary Premises for Undertaking Physical Activity

Freedom of choice, which characterizes free time activities, is often limited by the individual features of a person, financial potential, place of living and living conditions, a range of cultural activities and real availability [1,2,3,4]. In the modern world, one of the criteria for assessing quality of life is the organization of everyday life. A professionally active person gives special importance to free time, especially its quantity and forms of spending. They depend largely on preferred lifestyle. Representatives of various scientific disciplines—due to demographic processes, economic and socio-cultural tendencies—have paid more and more attention to the issue of free time, which is equated with recreation and leisure [5,6,7,8,9,10]. Leisure time considered from a social point of view is determined by several factors. First of all, there is the cultural level of society, whose height affects the creation of conditions for valuable forms, the organization and arrangement of recreation centers, cultural centers, artistic institutions, giving the opportunity for useful and valuable leisure time and a negative attitude towards primitive forms [4]. Secondly, there is the factor of general access to the infrastructure of work centers, culture, sport, nutrition, etc., i.e., from communication conditions determining the budget of free time, as well as networks of commercial, service, cultural and recreational centers [4,6]. Separate factors affecting people’s free time are environmental factors, which include: the degree of concentration of the population (the greater, the better conditions for organizing free time), the occupational diversity of residents (the greater, the richer the range of needs related to free time), conditions material (the higher the level of economic development, the better the organization of leisure time institutions and institutions), the activities of social and cultural organizations (the intensity of organization of free time depends on the creativity and activity of social and cultural organizations), an adequate number of places, centers adapted to organize recreational activities, their equipment and preparation of instructors, the tradition of spending time in a given region, the rational urbanization of housing estates (parks, squares, proximity of the forest), the state of education, and upbringing [11,12,13,14,15,16,17]. The tendencies of contemporary civilization specifically affirm health. It is recognized as an autotelic value that is important to every individual and to society as a whole. Health absolutely correlates with physical activity. The results of many studies suggest that in the majority of the examined youth, neither the family nor the school managed to instill the habit of spending free time actively [17,18,19,20].

According to many authors, health seems to be the most important motivating factor for participation in physical activity [21,22,23,24,25]. Consistent physical activity is the way to maintain the physical functioning of the body and reduces risks for chronic diseases [20]. Unfortunately, our care for health and well-being is definitely insufficient to maintain and improve basic health parameters [26,27]. This is the reason for the growing interest of specialists in many fields in the area of free time, regardless of age, gender or level of physical activity [28,29,30], and the difficulties and barriers that contribute to the decline in physical activity of societies and the availability of sports and recreation infrastructure [31,32,33,34].

Mass media, mass sports and recreational events dedicated to amateur athletes play an important role in the process of health and active lifestyle promotion. It is an important element of the social policy of many countries, including Poland [35,36,37,38]. On the other hand, the assessment of the involvement of local authorities in creating conditions for physical activity of residents ranked Poland in last place [39]. Poles are still the less active inhabitants of southern European countries (including Greece), compared to the inhabitants of Scandinavian countries, where the vast majority of citizens are active [40]. On the other hand, a turn towards a healthy and active lifestyle has been observed in Poland for a long time. The manner of consumption and the chosen forms of physical activity are changing, among the physically active Poles [41]. In this respect, Poles are heading towards western patterns [35]. According to Eurobarometer, only 27% of citizens perform regular activities in Poland and this result puts the country in one of the lowest positions in the EU [42]. According to a CBOS report, the main motive for undertaking sport by Poles is health (70%), followed by pleasure (61%) [43].

The research conducted as part of Social Project 2012 in Poland, commissioned by the Ministry of Sport and Tourism, shows that there was observed a significant variation in the frequency of physical activity by the inhabitants of individual voivodeships. Its results show that only in two voivodeships, Greater Poland and Lower Silesia, was the share of active people (doing sport every day, or often) exceeded the share of the inactive [44]. The analytical report Evaluation of the Social Benefits of Investment in Sport in Relation to the Costs Incurred carried out in 2016 by the Ministry of Sport and Tourism shows that young people are much more often active than the elderly. The level of activity and intensity decreases with age. In the 15–24 age group, the percentage of those with the lowest level of physical activity is 38%, whereas in the 55–64 age group, it is 72%; it is 95% in the 75–84 age group. On the other hand, the physically inactive are 24%, 61% and 91% of respondents, respectively. Similarly, the percentage of those with the highest level of physical activity decreases with age. Among those aged 15–24, they constitute 39% of the population, while in the 55–64 age group they account for 12%. In addition to age, another factor significantly differentiating the level of physical activity is education, where it can be observed that the percentage of physically active in the group with higher education is higher and amounts to 53%. The average time devoted to physical activity is 2 h and 50 min weekly [40]. These data are confirmed by the CBOS report The Physical Activity of Poles published by Małgorzata Omyła-Rudzka in 2013 [43]. The favorite disciplines of Poles are running (33% of the physically active), swimming (29%) and cycling (53%) [45], and the last two disciplines require special facilities to participate in.

### 1.2. Factors Limiting Access to Physical Activity

The benefits of regular physical activity help to improve overall health and fitness, maintain a healthy weight, reduce the risk for many chronic diseases and premature mortality and promote good mental health [46,47,48]. Unfortunately, people experience a variety of personal and environmental barriers to engaging in regular physical activity. The most common reasons that adults do not adopt more physically active lifestyles are cited as [49,50,51]: insufficient time to exercise, inconvenience of exercise, lack of self-motivation, non-enjoyment of exercise, boredom with exercise, lack of confidence in their ability to be physically active (low self-efficacy), fear of being injured or having been injured recently, lack of self-management skills (such as the ability to set, monitor, progress toward, or reward progress toward personal goals); lack of encouragement, support, or companionship from family and friends; non-availability of parks, sidewalks, bicycle trails, or safe and pleasant walking paths close to home or the workplace. According to Manaf, the top three barriers to engaging in physical activity across the adult lifespan are [51]: time, lack of energy and motivation. Authors dealing with the issue of barriers in undertaking physical activity list such factors as [52,53,54,55,56,57,58,59,60]: demographic, health-related, behavioral, cognitive, psychological, social and environmental (see Table 1).

It is obvious that the environment in which we live has a great influence on our level of physical activity [19,58], and many factors including the accessibility of walking paths, cycling trails, and recreation facilities affect us. Factors such as traffic, availability of public transportation, crime, and pollution may also have an influence on quality of life. Factors including the social environment, such as support from family and friends, and community spirit are also important [57,61]. Some of the barriers to physical activity that people face include family responsibilities, as well as cultural or social beliefs, economic or employment status, level of education, lack of time, lack of motivation, lack of energy, lack of money, health conditions, parenting demands, gender stereotyping, limited mobility and cultural expectations, which may restrict the participation of (especially) women in certain forms of physical activity [20,61,62,63,64,65,66,67].

Therefore, an attempt was made to analyze the causes for the low level of physical activity in the society, as its importance to health has been recognized. Attention was paid to what main barriers in undertaking physical activity were indicated by respondents in surveys conducted in Europe by the Eurobarometer, in Poland by the Central Statistical Office (GUS), and in Poznan by means of our own research. The basic reason for failing to undertake physical activities is the time deficit—the majority of Poles justify their low physical activity with the lack of free time. However, as Kocemba [68] has noted, indicating the lack of time as an objective obstacle to participate in physical culture is a classic rationalization of views and the concealment of more authentic reasons for preferring another type of activity. Another constraint that hinders regular physical activity is poor health, illness or disability, which often precludes from doing sports. Furthermore, research indicates reluctance of some people to undertake any types of exercise [42]. The Eurobarometer results from 2017 show that 39% of surveyed Poles admitted that they were not interested in being physically active and that they would like to engage in other forms of activity. The results also underlined that infrastructure plays an important role in taking up regular sport activities—insufficiently developed sports facilities and lack of access to sports and recreation facilities is a barrier that is especially prominent in rural areas. Moreover, socio-economic limitations and high costs of participation in some forms of activity are indicated. As a result, some of the facilities are available only to people with relatively high incomes. On the other hand, it should be noted that the majority of people who declare exercising on a regular basis do it outdoors, in parks. Furthermore, more than half of the respondents of Eurobarometer in 2017 agreed with the statement that in their place of residence the conditions necessary for doing sports are provided [42]. The argument about the lack of adequate access to infrastructure or high costs of using sports facilities and equipment is not always justified.

Poznan is a city located in West-Central Poland. The city’s population is about 540,000. It is an important academic site (with about 140,000 students), a center of trade, sports, technology, and tourism. It has often topped rankings as a city with very high-quality education and a very high standard of living [69]. It also ranks highly in safety and quality of healthcare [70]. Sports and recreation are important areas of activity for the city. Each year, more than 10% of Poznan’s budget is spent on physical culture, which is a level comparable to that of the European Union average [71]. Poznan’s expenditures allocated to physical culture are the highest in the country and account for PLN 410 million, which is 12.3% of the annual budget of the city. Only Warsaw, which also allocates PLN 410 million a year for this purpose, can compete with Poznan, but this amount is only 3.3% of the capital’s budget [72]. Poznan undoubtedly strives to be a city that “puts on sport”. In the resolution of the City Council adopted in 2010, “Strategy for the City of Poznan until 2030,” one of the strategic goals is to increase the importance of the city as a center of sport. Poznan has over 500,000 residents [72]. The sports and recreation infrastructure is very complex because it consists of various elements, from huge recreational complexes to modern large-area clubs to intimate places associated with the chosen form of recreation. The diverse offer of sports and recreation facilities proposed by the city for its residents undoubtedly translates into a better image for these places. That is, the residents’ perception of the territorial unit and opinion of it is more likely to be positive [72].

## 2. Materials and Methods

### 2.1. Research Design and Data Collection

The main goal of this study is to recognize difficulties limiting the access to sports and recreation facilities in the city of Poznan.

The research carried out at indoor tennis courts (16 facilities, 480 respondents), indoor swimming polls (12 facilities, 360 respondents) and fitness clubs (11 facilities, 319 respondents) in Poznan covered 1159 service recipients (using the services of a given sports and recreation facility) (Table 2). The self-constructed questionnaires were addressed to the service recipients (residents of the city of Poznan, who use the services offered at the examined sports and recreation facilities) and was validated in selected sports facilities in Poznan, six months before the main study. After verification and evaluation of the effectiveness of the research tool, a targeted selection of respondents was conducted in selected all-season sports and recreation facilities among 1159 service recipients (using the services of a given sports and recreation facility). Furthermore, the service recipients were divided into a group of people doing sports competitively and a group of recipients who have never practiced sports as professional athletes. These questionnaires were conducted by the authors among service recipients in the sports and recreation facilities in the areas of tennis courts, indoor swimming pools and fitness clubs and were personally filled out during a conversation with the recipients.

The first part of the questionnaires focused on socio-demographic variables like age and education level (Table 1, Table 2 and Table 3). The second part of questionnaire was strictly connected with the sports and recreation facilities in Poznan and was related to their availability and quality assessment. Referring questions to people using the examined objects was purposeful and justified. The respondents’ opinions were based on their own experience and therefore reliable. The survey was intentionally addressed to people who use sports and recreation facilities to find out their opinion on any existing difficulties in their use of facilities. The authors of the study wanted the survey results to show not why people do not use the object, but what makes it difficult for them to use it. The information obtained may constitute the basis for proposing changes in the functioning of the examined objects and in the city’s policy regarding, for example, the development of communication infrastructure. Changes in the functioning of sports and recreational facilities and changes in the population (society, people using these facilities) force this type of research to be repeated periodically. This type of research was not conducted in the city of Poznan in sports and recreation facilities before. Research using standardized interview made it possible to collect primary data concerning the presentation of the pace and directions of changes (trends) taking place in the sports and recreation facilities of the city. The presented results are a part of a large research project regarding sports and recreational infrastructure management policy of Poznan.

### 2.2. Data Analysis

In the questionnaire, the surveyed group of respondents could mark YES or NO answers, as well as express their level of satisfaction/evaluation with a grading scale from 1 to 10 (1—unsatisfied; 10—very satisfied). The following research methods were used to conduct the analysis of the studied phenomena: descriptive statistics, histograms, testing equality of means (ANOVA), testing equality of variance (Bartlett test). The study tested means for more than two variables, therefore the one-way ANOVA procedure was chosen for this purpose. The zero hypothesis of the equality test is:

$$H_{0}:{\bar{X}}_{1}={\bar{X}}_{2}=\ldots ={\bar{X}}_{k}$$

where H_0_ represents zero hypothesis, $\bar{X}$ represents arithmetic mean and $\bar{X}$ represents k-number of series.

The test statistics have an F-Snedecor asymptotic distribution with a number of degrees of freedom equal to g – 1 and T – g (where g is the number of populations, in this case the number of assessment groups from 1 to 10). As in the case of ANOVA, equality of variance was tested for more than two variables. The Bartlett test used, which has a zero hypothesis:

H_0_: *σ*_1_^2^ = *σ*_2_^2^ =… *σ*_k_^2^

where H_0_ represents the null hypothesis and σ2k represents the k-number of series distribution of normal results.

Empirical probabilities (*p*-values) were calculated using distributions of test statistics. The test results reporting tables present test statistic values and empirical probabilities. Values of empirical probabilities lower than the assumed level of significance (equal to 0.1) indicate the rejection of the null hypothesis about the equality of means (or variance).

### 2.3. Participants

The recipients were mainly at the age of 20–29 years old (49.3%; *n* = 572) and 30–39 years old (24.2%; *n* = 281). Among the surveyed people, the minority were aged 50 years old and older (3.6%; *n* = 42) (Table 4).

People with higher education constituted the vast majority of respondents at 55% (637). A total of 30.7% (356) possessed secondary education, and 14.3% (166) were people with primary education. The respondents who were professionally involved in sport comprised 41% (476), and those who never practiced sport competitively comprised 59% (683).

## 3. Research Results

**Figure 1:**
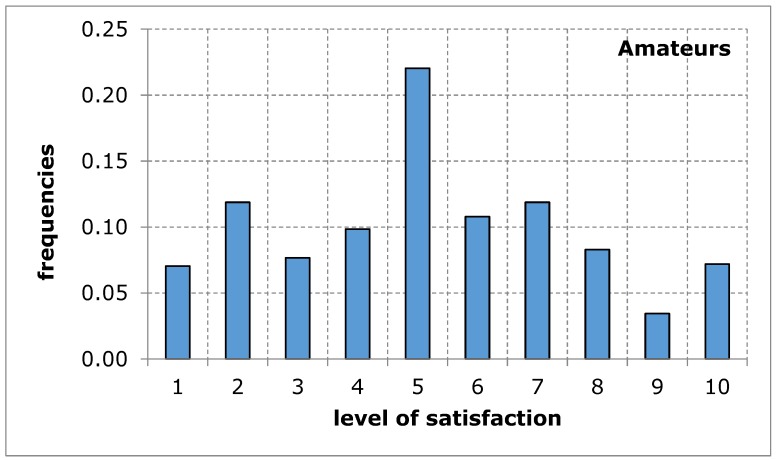
A sports and recreation facility is not publicly available to residents of the city of Poznan, in the opinion of service recipients (Amateurs). Source: own work.

**Figure 2:**
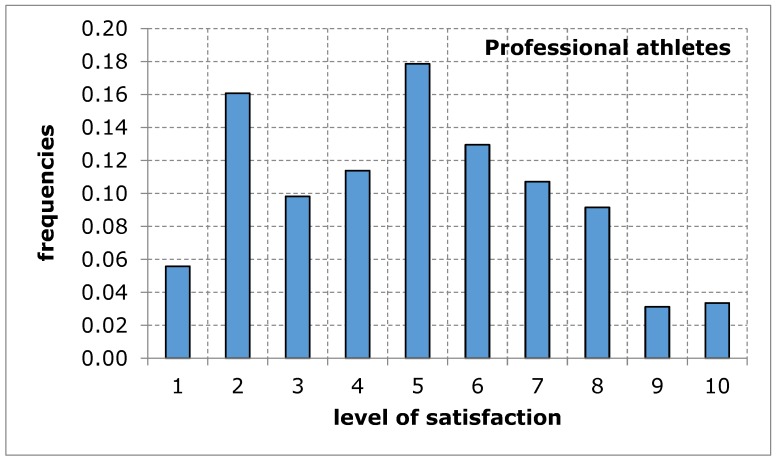
A sports and recreation facility is not publicly available to residents of the city of Poznan, in the opinion of service recipients (professional athletes). Source: own work.

The questionnaire addressed to the service recipients included suggestions of difficulties that may limit the availability of their use of sports and recreation facilities in the city of Poznan. When evaluating the availability of sports and recreation facilities by a group of amateurs and professional athletes, similar results were obtained in both groups of recipients. The distributions of the variables tested in terms of the measures of positions, variation and description of distribution shape do not differ from each other, which is confirmed by tests of mean and variance equality, despite relatively low probability values: 9.3% for mean and 13.4% for variance (Table 5). Empirical distributions resemble, at least for some ranges of responses, a uniform distribution, for which the probability of obtaining appropriate values is equal (Figure 1 and Figure 2). The obtained research results indicate an average level of satisfaction of the surveyed service recipients with the availability of sports and recreation facilities in the city of Poznan. This average level of satisfaction with the availability of sports and recreation facilities can largely weaken sports and recreational activity of the residents of the city of Poznan. Investigating the importance of barriers limiting the availability of sports and recreation facilities in the further course of the analysis will eventually indicate the reason for the recreational activity of residents being limited.

**Figure 3:**
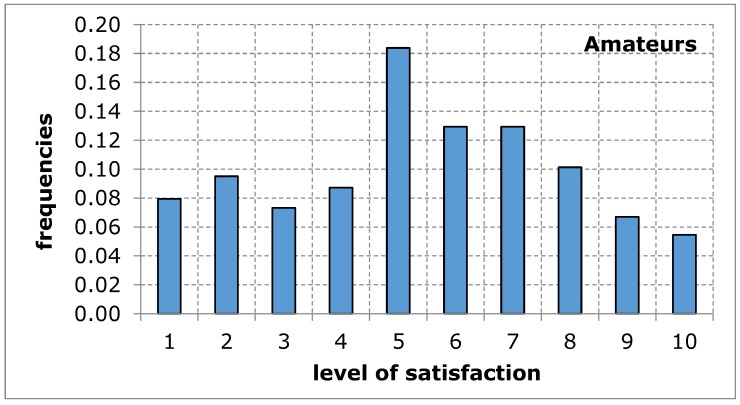
A sports and recreation facility is too far away, in the opinion of service recipients (amateurs). Source: own work.

**Figure 4:**
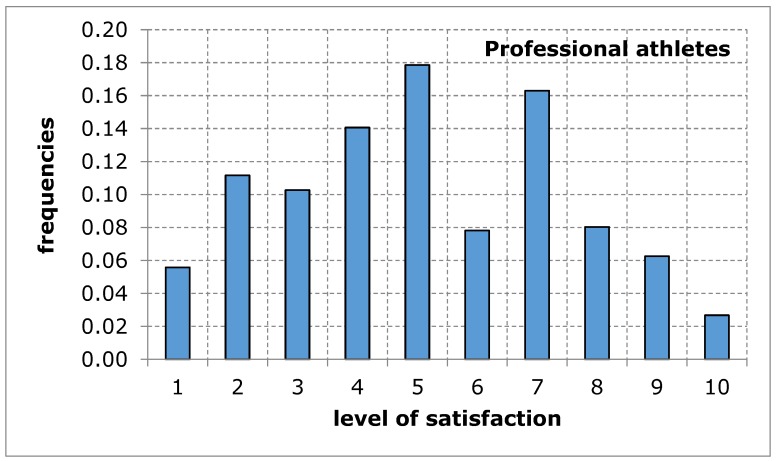
A sports and recreation facility is too far away, in the opinion of service recipients (professional athletes). Source: own work.

As far as the assessment of the availability of a sports and recreation facility from the point of view of the distance from the place of residence was concerned, the opinion of both groups of respondents was similar in terms of the average value. The shape of the distribution of the variable under study in both groups shows a tendency of left-skewness, thus indicating a lower level of satisfaction. The obtained results suggest that the distance of the sports and recreation facility from the place of residence may constitute a difficulty in the accessibility of the facility, thus limiting the possibility for the city’s residents to carry out recreational activities (Figure 3 and Figure 4, Table 6).

**Figure 5:**
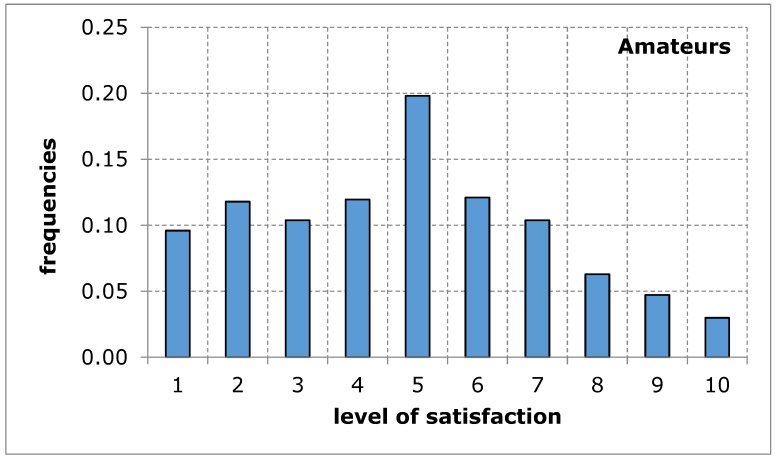
Difficult access to a sports and recreation facility, in the opinion of service recipients (amateurs). Source: own work.

**Figure 6:**
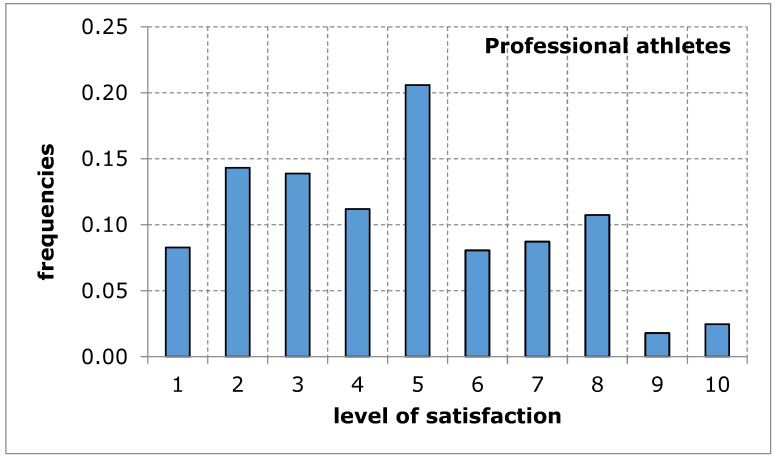
Difficult access to a sports and recreation facility, in the opinion of service recipients (professional athletes). Source: own work.

The respondents’ answers regarding transport barriers do not clearly define their level of satisfaction. The average values are similar in both groups. The distributions of the studied variables in terms of the measures of position, variation and description of distribution shape do not differ from each other, which is confirmed by the tests of equality of means and equality of variance. However, an analysis of the Figure 5 and Figure 6 allows assuming that the access to sports and recreation facilities is not significantly hindered for the customers. This is confirmed by statistical characteristics: positive values of the skewness coefficient, which, although not extremely high, point to the right-skewness, i.e., a high chance of achieving a score lower than the average. In fact, in the opinion of the service recipients, there are no major difficulties in getting to the facility (Table 7).

The level of satisfaction with prices at sports and recreation facilities has been determined as average (very similar average values) by both the group of amateurs and professional athletes (Table 8).

**Figure 7:**
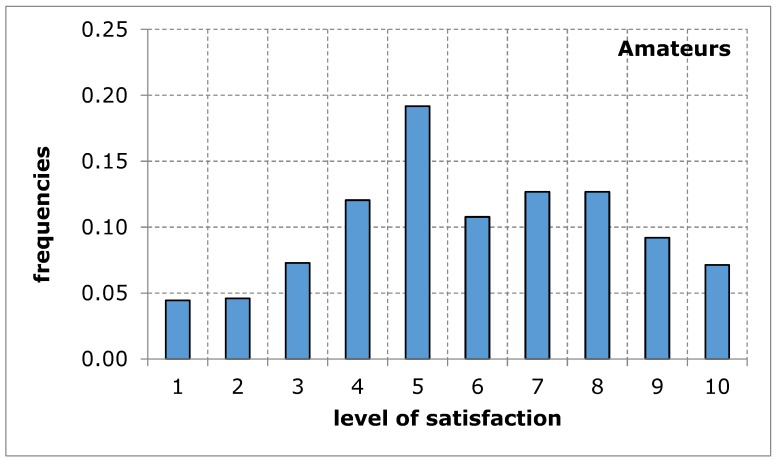
Relatively high prices of services at sports and recreation facilities, in the opinion of service recipients (amateurs). Source: own work.

**Figure 8:**
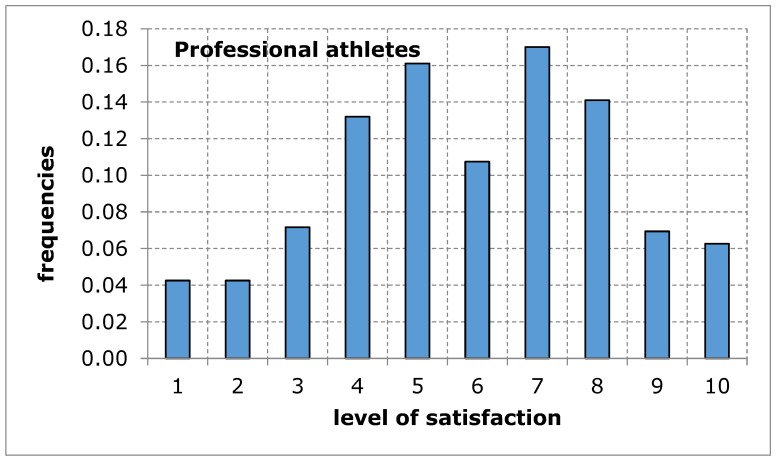
Relatively high prices of services at sports and recreation facilities, in the opinion of service recipients (professional athletes). Source: own work.

However, the shape of the distribution of the tested variable (negative values of the skewness coefficient) indicates, especially in the group of professional athletes, the tendency to evaluate the prices at sports and recreation facilities as too high, which leads to the conclusion that this is a significant barrier to performing sports and recreational activities at sports and recreation facilities in the city of Poznan (Figure 7 and Figure 8).

**Figure 9:**
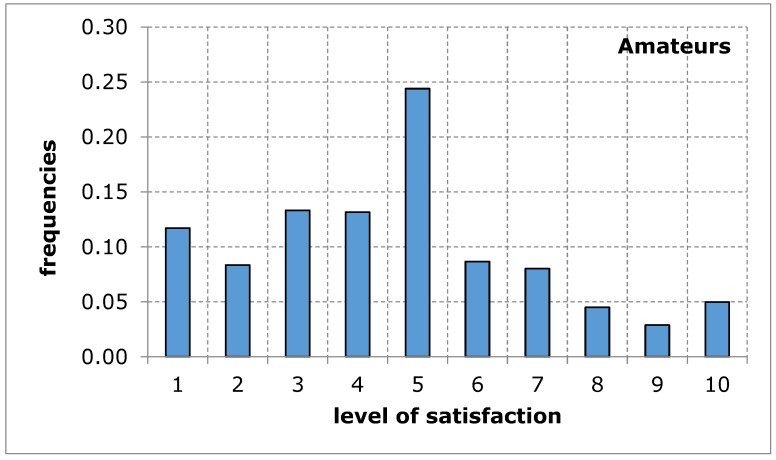
Use of a sports and recreation facility for competitive sport purposes, in the opinion of service recipients (amateurs). Source: own work.

**Figure 10:**
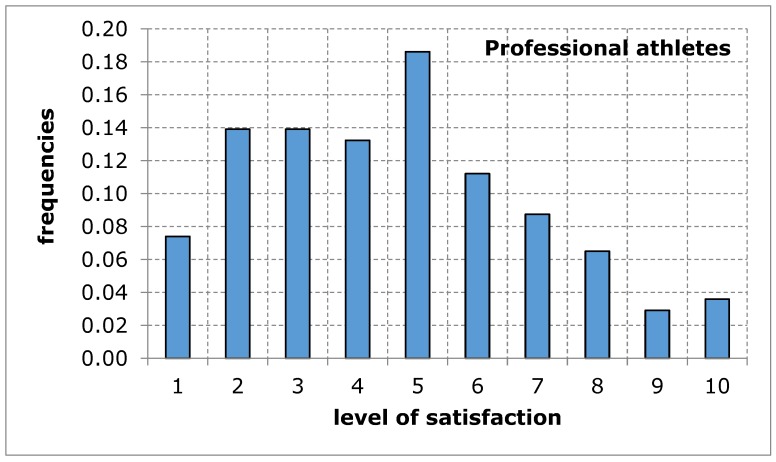
Use of a sports and recreation facility for competitive sport purposes, in the opinion of service recipients (professional athletes). Source: own work.

In the assessment of the use of a sports and recreation facility for competitive sport purposes, the service recipients showed an average level of satisfaction in both groups of respondents. This is evidenced by almost identical mean values (Table 9 and Figure 9). However, the analysis of the description of distribution shape of the variables studied indicates that professional athletes do not consider the assessed facilities to be particularly adjusted to competitive sport, and therefore it can be concluded that sports and recreation facilities in the city of Poznan offer similar opportunities for sporting and recreational activities, done both by amateurs and professional athletes (Figure 10).

**Figure 11:**
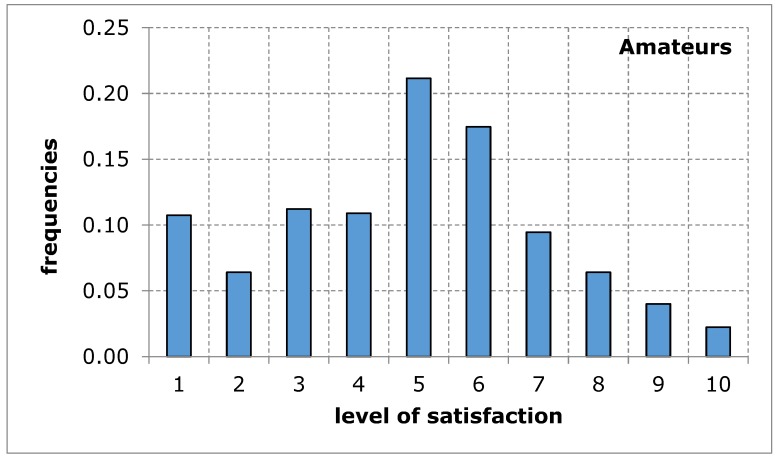
Lack of preferred sport and recreational activities at a sports and recreation facility, in the opinion of service recipients (amateurs). Source: own work.

**Figure 12:**
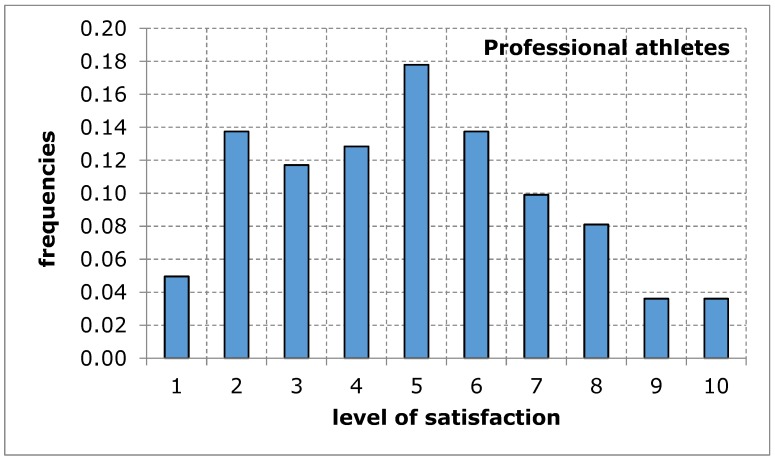
Lack of preferred sport and recreational activities at a sports and recreation facility, in the opinion of service recipients (professional athletes). Source: own work.

As far the assessment of the offer of additional services at sports and recreation facilities is concerned, the service recipients indicated, in both groups of respondents, a level of satisfaction that is lower than the average. Differences between means, similarly to variance (Table 10), are statistically insignificant. It can be said with rather moderate probability that both studied groups perceive this problem in the same way. However, the probability distributions differ due to skewness signs, as a greater number of low ratings are significantly more visible among the professional athletes (Table 10). This proves that the service recipients have observed a partial lack of preferred activities at sports and recreation facilities that they use. It is worth emphasizing the fact that a higher level of dissatisfaction with the offer of classes has been demonstrated by the service recipients who are professional athletes. Thus, it may be assumed that they have higher expectations towards the offer of sports and recreation facilities (Figure 11 and Figure 12).

## 4. Conclusions

It is obvious that consistent physical activity helps to maintain the physical functioning of the human body, improves mental wellbeing [26] and most of all reduces risks for chronic diseases [26]. Given these widely published benefits, one would expect participation in physical activity to be the norm. Unfortunately, this is not the case. What is the reason? What is the difficulty? The summary of the analysis of the opinions of service recipients, broken down into amateur and competitive sports practitioners, about the attractiveness of sports and recreation facilities, the possibilities of using them, difficulties limiting accessibility to them and the impact of the Poznan City Hall policy on their functioning has provided interesting conclusions. Changes in the functioning of sports and recreational facilities and changes in the population (society, people using these facilities) force this type of research to be repeated periodically.

The presented studies are innovative and do not have significant support in the literature. There are many studies on PA barriers among non-exercisers, but there are no results presenting the research conducted among people who use sports and recreation facilities. These studies can help to find out their views on any existing difficulties in their use of facilities. The best example and reference point for our research is the most recent edition of The Geography of Tourism and Recreation (2014), 4th Routledge, by Hall and Page [73], as it has a substantial discussion of recreation and leisure constraints with respect to recreation and sports facilities. This is an important book in the field of tourism, leisure and recreation from geographical and social science perspectives. The authors show important barriers that concern participation in recreation and in broadly understood tourist activity. It still remains the only book to systematically compare and contrast, in a spatial context, tourism and recreation in relation to leisure time, offering insight into the demand, supply, planning, destination management and impacts of tourism and recreation [73]. This type of research has not been conducted in the city of Poznan in sports and recreation facilities before. Research using standardized interview made it possible to collect primary data concerning the presentation of the pace and directions of changes (trends) taking place in the sports and recreation facilities of the city of Poznan. Both groups of respondents highly rated the level of equipping sports and recreation facilities as well as the availability of services and the offer of sports and recreation activities. A lower level of satisfaction and thus a lower rating of the respondents was obtained by a set of features constituting, according to the respondents, difficulties limiting the access to the use of sports and recreation facilities (high prices and too great of a distance of the sports and recreation facility from the place of residence). Therefore, they clearly constitute obstacles to the implementation of recreational activities for the inhabitants of the city of Poznan. The assessment of the policy of the city authorities of Poznan, the test of significance of variance of the studied variables, between amateur and competitive sports practicing groups, also showed an unfavorable difference in the distribution of the examined features, thus reflecting the diversity of opinions in the examined groups of recipients. These differences most likely result from the different expectations of the surveyed people practicing sports and amateur using sports and recreation facilities in view of the effectiveness of the policy of the Poznan City Hall. Therefore, it should be varied and properly adapted to the requirements, type and nature of the expectations of recipients, different depending on the intensity of their use of the sport and recreation services offered to them.

Only about 4% of residents of cities of over 500,000 citizens report the lack of access to infrastructure facilities that can be used to do physical recreation near the place of residence [74]. According to a report carried out at the request of the Ministry of Sport and Tourism in 2017 [75], residents of Wielkopolska assessed the number and availability of sports and recreation facilities in their region high, with 69.5% stating that “they are just right” and 60% saying they are in good conditions. The surveyed service recipients assessed the lack of accessibility and distance to sports and recreation facilities in Poznan to be less important than the financial barrier, which they placed at the first place.

Research on the impact of the type of sports and recreation infrastructure and its availability was carried out in 2009 for Stuttgart, Germany [76]. The research has introduced an additional element, which is the problem of the availability of sports and recreation infrastructure adequate for individual age groups of city residents. Although the research on this problem is incomplete, it shows that an important barrier to physical activity of residents is both the lack of knowledge about the existing sports and recreation infrastructure and the distance to it. It is known that participation in sports and recreational activities changes over the course of a person’s life. As a result, particular types of sports and recreation facilities are used only by some age groups, whereas for others, they are of little importance, e.g., for people aged 36–64, good accessibility of sports and recreation infrastructure is of little importance, while the opening hours are more significant [76]. Although the results of research conducted for Stuttgart are exemplary, they can be an important clue in the interpretation of the relationship between infrastructure and participation in sports and recreation activities of city residents.

Due to the low level of physical activity of our society and the existing barriers and limitations, it is necessary to undertake actions aimed at improving the situation and ensuring financial support by public administration bodies, both state and local government.

Among the European Union guidelines concerning physical activity, the most important are access to sports and recreation infrastructure [77] and, most significantly, free or inexpensive use of public sports and recreation facilities. The authorities of the city of Poznan should follow such guidelines when developing sports and recreation facilities and promoting a healthy, active lifestyle of residents. However, it should be remembered that the size and condition of the sports and recreation infrastructure depends primarily on the level of the country’s economic development and pro-health awareness of its citizens [78,79,80,81]. Nobody has studied the difficulties limiting the access to sports and recreation facilities in the city of Poznan. The survey was intentionally addressed to people who use sports and recreation facilities to find out their opinion on any existing difficulties in their use of facilities. The results indicated those elements of the functioning of the facilities that can be improved and thus increase the number of people using the facilities in the researched city in the future. Similar surveys should be carried out among Poznan residents who do not declare their willingness to use sports and recreation facilities.

## Figures and Tables

**Table 1:** Factors limiting undertaking physical activity.

Factors	Notes
Demographic Factors	- Older age: poor health status, poor perception of overall health, depressive symptoms, mobility limitations, pain and fear of pain, lower self-efficacy, lower social support, small adaptive beliefs and lack of encouragement from physicians; - Gender: family responsibilities, household duties, lack of time, gender stereotyping, limited mobility and some cultural expectations restrict the participation of women in PA; - Low socioeconomic background (SES): lack of money, lack of facilities in the community, lack of social support, lack of work flexibility.
Health-Related Factors	- Poor general health and physical function, obesity, chronic illnesses
Behavioral Factors	- Prior PA, smoking, drinking alcohol, negative social influence
Cognitive and Psychological Factors	- Greater perceived barriers to physical activity: lack of time, lack of facilities, fatigue, poor health and self-consciousness about appearance; - Lack of enjoyment of physical activity: low expectations of benefits from physical activity, poor psychological health; - Low self-efficacy for physical activity, i.e., someone’s confidence in their ability to be physically active in a regular basis; - Low self-motivation for physical activity; - Poor fitness level.
Social Factors	- Lack of cohesion in exercise group, lack of physician influence/advice for physical activity, lack of social support for physical activity.
Environmental Factors	- Lack of access to facilities/parks/trails, as inconvenient access to facilities may lead to lower adherence to PA; - Lack of neighborhood safety, as neighborhood safety is particularly important among women, older adults, and individuals with lower education level.

Source: own study based on literature. PA: Physical Activity.

**Table 2:** Characteristics of respondents.

Age Characteristics of the Respondents	Tennis Courts (*n* = 480)		Indoor Swimming Pools (*n* = 360)		Fitness Clubs (*n* = 319)		All (1159)
	n	%	n	%	n	%	
<20	78	17.3	16	7.8	92	17.5	186
20–29	225	49.8	99	48.5	248	47.1	572
30–39	96	21.2	67	32.8	118	22.4	281
40–49	35	7.7	13	6.4	54	10.2	102
50+	18	4.0	9	4.4	15	2.8	42

**Table 3:** Descriptive statistics-for observations from sample *n* = 1159 for the Age variable.

**Mean**	**Median**	**Minimum**	**Maximum**
27.7990	25.0000	15.0000	67.0000
**Standard Deviation**	**Coefficient of Variation**	**Skewness**	**Kurtosis**
9.66253	0.347586	1.20478	1.52895

**Table 4:** Descriptive statistics for observations from sample *n* = 1159 for the age variable in each object.

	**Mean**	**Median**	**Minimum**	**Maximum**
Indoor swimming pools	27.9624	25.0000	15.0000	67.0000
Tennis courts	29.3676	27.0000	15.0000	60.0000
Fitness clubs	27.7780	25.0000	16.0000	56.0000
	**Standard Deviation**	**Coefficient of Variation**	**Skewness**	**Kurtosis**
Indoor swimming pools	10.4107	0.372310	**1.42351**	**2.25329**
Tennis courts	8.93860	0.304369	0.806148	0.216468
Fitness clubs	9.08693	0.327127	0.829227	−0.00652774

**Table 5:** Statistical characteristics of the availability of sports and recreation facilities for the residents of the city of Poznan, in the opinion of service recipients.

Statistical Measures	$\bar{X}$	S	SK	K	N	Test for Equality of Means *p*	Test for Equality of Variances *p*
Amateurs	4.874	2.729	0.031	2.253	683	0.093	0.134
Professionals	4.607	2.563	0.072	2.231	476		

Source: own work.

**Table 6:** Statistical characteristics of the availability of sports and recreation facilities for the residents of the city of Poznan, in terms of place of residence.

Statistical Measures	$\bar{X}$	S	SK	K	N	Test for Equality of Means *p*	Test for Equality of Variances *p*
Amateurs	5.072	2.760	−0.137	2.137	683	0.123	0.126
Professionals	4.824	2.588	−0.038	2.198	476		

Source: own work.

**Table 7:** Statistical measures of difficult access to a sports and recreation facility, in the opinion of service recipients.

Statistical Measures	$\bar{X}$	S	SK	K	N	Test for Equality of Means *p*	Test for Equality of Variances *p*
Amateurs	4.508	2.666	0.288	3.033	683	0.281	0.172
Professionals	4.340	2.518	0.179	2.254	476		

Source: own work.

**Table 8:** Statistical characteristics of the relatively high prices of services at sports and recreation facilities, in the opinion of service recipients.

Statistical Measures	$\bar{X}$	S	SK	K	N	Test for Equality of Means *p*	Test for Equality of Variances *p*
Amateurs	5.436	2.788	−0.292	2.312	683	0.569	0.2758
Professionals	5.529	2.663	−0.363	2.436	476		

Source: own work.

**Table 9:** Statistical characteristics of a sports and recreation facility for competitive sport purposes, in the opinion of service recipients.

Statistical Measures	$\bar{X}$	S	SK	K	N	Test for Equality of Means *p*	Test for Equality of Variances *p*
Amateurs	4.223	2.627	0.260	2.516	683	0.3687	0.3367
Professionals	4.361	2.523	0.237	2.434	476		

Source: own work.

**Table 10:** Statistical characteristics of the lack of preferred sports and recreational activities at a sports and recreation facility, in the opinion of service recipients.

Statistical Measures	$\bar{X}$	S	SK	K	N	Test for Equality of Means *p*	Test for Equality of Variances *p*
Amateurs	4.452	2.570	−0.073	2.284	683	0.3395	0.9012
Professionals	4.599	2.557	0.082	2.332	476		

Source: own work.
